# Evaluation of Three Portable Optical Sensors for Non-Destructive Diagnosis of Nitrogen Status in Winter Wheat

**DOI:** 10.3390/s21165579

**Published:** 2021-08-19

**Authors:** Jie Jiang, Cuicun Wang, Hui Wang, Zhaopeng Fu, Qiang Cao, Yongchao Tian, Yan Zhu, Weixing Cao, Xiaojun Liu

**Affiliations:** 1 National Engineering and Technology Center for Information Agriculture, Nanjing Agricultural University, Nanjing 210095, China; 2019201009@njau.edu.cn (J.J.); 2019201008@njau.edu.cn (C.W.); 2019101173@njau.edu.cn (H.W.); 2018801234@njau.edu.cn (Z.F.); qiangcao@njau.edu.cn (Q.C.); yctian@njau.edu.cn (Y.T.); yanzhu@njau.edu.cn (Y.Z.); caow@njau.edu.cn (W.C.); 2 MOE Engineering Research Center of Smart Agricultural, Nanjing Agricultural University, Nanjing 210095, China; 3 MARA Key Laboratory for Crop System Analysis and Decision Making, Nanjing Agricultural University, Nanjing 210095, China; 4 Jiangsu Key Laboratory for Information Agriculture, Nanjing Agricultural University, Nanjing 210095, China; 5 Jiangsu Collaborative Innovation Center for Modern Crop Production, Nanjing Agricultural University, Nanjing 210095, China

**Keywords:** nitrogen indicator, nitrogen nutrition diagnosis, optical sensor, spectral index

## Abstract

The accurate estimation and timely diagnosis of crop nitrogen (N) status can facilitate in-season fertilizer management. In order to evaluate the performance of three leaf and canopy optical sensors in non-destructively diagnosing winter wheat N status, three experiments using seven wheat cultivars and multi-N-treatments (0–360 kg N ha^−1^) were conducted in the Jiangsu province of China from 2015 to 2018. Two leaf sensors (SPAD 502, Dualex 4 Scientific+) and one canopy sensor (RapidSCAN CS-45) were used to obtain leaf and canopy spectral data, respectively, during the main growth period. Five N indicators (leaf N concentration (LNC), leaf N accumulation (LNA), plant N concentration (PNC), plant N accumulation (PNA), and N nutrition index (NNI)) were measured synchronously. The relationships between the six sensor-based indices (leaf level: SPAD, Chl, Flav, NBI, canopy level: NDRE, NDVI) and five N parameters were established at each growth stages. The results showed that the Dualex-based NBI performed relatively well among four leaf-sensor indices, while NDRE of RS sensor achieved a best performance due to larger sampling area of canopy sensor for five N indicators estimation across different growth stages. The areal agreement of the NNI diagnosis models ranged from 0.54 to 0.71 for SPAD, 0.66 to 0.84 for NBI, and 0.72 to 0.86 for NDRE, and the kappa coefficient ranged from 0.30 to 0.52 for SPAD, 0.42 to 0.72 for NBI, and 0.53 to 0.75 for NDRE across all growth stages. Overall, these results reveal the potential of sensor-based diagnosis models for the rapid and non-destructive diagnosis of N status.

## 1. Introduction

Nitrogen (N) is an essential nutrient that improves crop growth and grain yield. The excessive application of N fertilizers can lead to low N use efficiency, resulting in environmental pollution and a loss of grain quality [1,2]. Precision N management could be used to optimize N application by considering the temporal and spatial variability of crop N status in practical production [3,4]. However, this promising strategy requires the development and application of real-time and non-destructive technologies for in-season crop N nutrition diagnosis.

Leaf and plant N concentrations (LNC, PNC) have been used as vital parameters of crop N status [5]. However, N concentrations are dependent on the plant biomass, such that two different plants with the same N concentration but differ in plant biomass. The critical N concentration dilution curve (CNDC) reflects the power–function relationship between crop critical N concentrations and plant biomass [6]. Based on the CNDC, the N nutrition index (NNI) could be calculated to effectively diagnose crop N nutrition status [7]. For example, NNI values greater than one indicate excessive N status, while values less than one correspond to N deficiency. Previous studies indicated that the NNI diagnosis model has been successfully used for characterizing corn (R^2^ = 0.33–0.68), wheat (R^2^ = 0.73–0.86), and pepper (R^2^ = 0.19–0.84) N status throughout crop growth stages [8,9,10]. However, the calculation of NNI requires complicated chemical analysis to determine PNC. Plant biomass measurements using destructive sampling are also time-consuming and unsuitable for in-season N management [11,12].

The application of proximal and remote sensing technology can provide an efficient method for real time crop N status estimations [13]. Optical transmission measurements with a handheld SPAD-502 chlorophyll meter (SPAD meter) have been widely used for crop N nutrition assessments due to their portability, fast responses, and affordable cost [14,15]. However, SPAD are easily influenced by the crop growth stage and cultivar leaf structure, with over-fertilized plants being challenging to detect due to chlorophyll saturation. The Dualex 4 Scientific+ sensor (Dualex) is a portable leaf fluorescence sensor that measures Chl values through leaf transmittance at 710 and 850 nm and epidermal flavonoid (Flav) levels through the assessment of chlorophyll fluorescence induced by ultra-violet (UV) excitation at 375 nm, and further providing a Chl/Flav ratio, which is termed the N balance index (NBI) [16]. Many studies have demonstrated a prominent relationship between Dualex-based indices and different N parameters. For example, Dualex-based Chl (R^2^ = 0.49–0.90) were found to correlate with leaf chlorophyll concentrations in rice, wheat, corn, and soybean [17,18]. Zhang et al. [17] indicated a significant relationship between Flav measured by Dualex and rice LNC (R^2^ = 0.52–0.83), PNC (R^2^ = 0.56–0.76), and NNI (R^2^ = 0.68–0.82) across different growth periods. Dualex-based NBI was also successfully used to monitor N nutrition status in corn, wheat, and other crops [19,20,21]. Gabriel et al. [22] compared two different leaf-clip sensors (SPAD meter and Dualex) to estimate corn LNC and indicated similar performances, with R^2^ = 0.43–0.62 for the SPAD meter and R^2^ = 0.42–0.68 for Dualex. Lejealle et al. [23] indicated that N balance index (NBI), the ratio of chlorophyll to flavonols that measured through by Multiplex, had an improved and more stable correlation with turfgrass LNC than Chl readings alone. Consequently, it is necessary to assess the performance of spectral indices collected from two leaf-sensors for winter wheat N status assessments.

Canopy optical sensors can collect spectral data at the canopy level compared to leaf sensor measurements at the leaf scale. Passive canopy optical sensors, including ASD Fieldspec and Cropscan, have been successfully used to monitor crop growth and to assess N status [24,25]; however, their correct function requires strict environmental conditions, such as light intensity and measurement times [26]. Active sensors, such as RapidScan CS-45 (RS sensor), possess an internal lights source that ensures effective measurements in suboptimal environmental conditions. This can be used to collect crop canopy spectrum values at 670, 730, and 780 nm wavelengths, with two default vegetation indices (normalized difference red edge (NDRE) and normalized difference vegetation index (NDVI)) synchronously. The relationship between the vegetation indices derived from the RS sensor and crop growth status has been extensively studied for various crops, including wheat and soybean [27,28,29].

While the SPAD meter, Dualex, and RS sensor have been widely used for estimations of crop growth and N status, the comparative assessment of these three portable sensors to real-timely diagnose winter wheat N nutrition have not been studied. The aims of this study were: (a) to evaluate the performance of the six sensor-based indices (leaf level: SPAD, Chl, Flav, and NBI; canopy level: NDRE, NDVI) for non-destructive estimates of N status of winter wheat; (b) to establish winter wheat N diagnostic models based on optimum leaf and canopy sensor-based indices, respectively; and (c) to plot N diagnosis maps temporally and spatially across all growth stages. These results can improve the non-destructive diagnosis of crop N nutrition, and could be used to guide appropriate N management strategies.

## 2. Materials and Methods

### 2.1. Experimental Design

**Figure 1:**
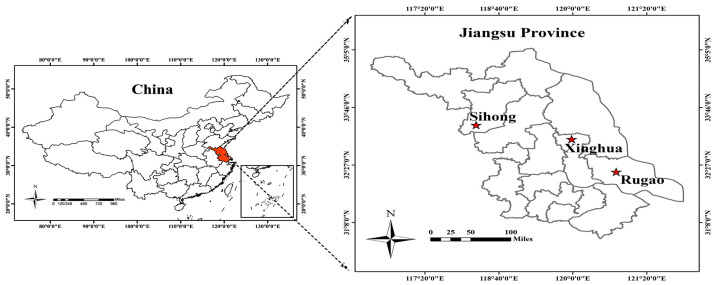
Three study sites in the Jiangsu province of China.

Experiment 1 (2015–2016), 2 (2016–2017), and 3 (2017–2018) were performed at the Sihong (Figure 1; 33.37° N, 118.26° E), Rugao (Figure 1; 32.27° N, 120.75° E), and Xinghua (Figure 1; 33.08° N, 119.98° E) Experimental Stations, respectively, in the Jiangsu province of China. All experiments were performed using different wheat cultivars and N application rates with three replicates in a randomized complete block design. Plants density was 225 seedlings per square meter. The N fertilizer (granular urea with 46% N) was applied in two batches: 50% prior to sowing and 50% at the stem elongation stage. Additionally, 105 kg ha^−1^ P2O5 and 135 kg ha^−1^ K2O were applied to all experiment plots. Detailed information is shown in Table 1.

### 2.2. Spectral Data Collection

**Figure 2:**
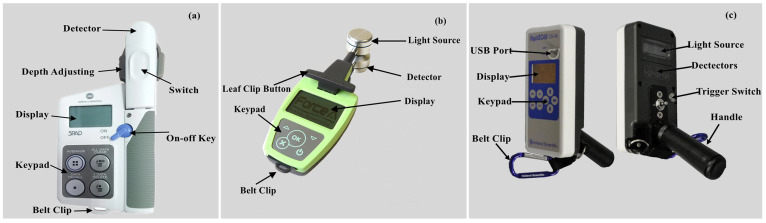
Images of the (**a**) SPAD-502 meter, (**b**) Dualex 4 Scientific+ sensor, and (**c**) RapidSCAN CS-45 sensor.

Three different optical sensors were used to collect wheat leaf and canopy spectral data at jointing, booting, flowering, and filling stages, respectively. The SPAD meter (Figure 2a; Minolta Camera Co., Osaka, Japan) and Dualex (Figure 2b; Dualex Scientific, Force-A Co., Orsay, France) were used to measure wheat leaf spectral parameters. The canopy sensor (Figure 2c; Holland Scientific Inc., Lincoln, NE, USA) was used to obtain wheat canopy spectral data. Detailed information of the three optical sensors is shown in Table 2.

The first, second, and third fully expanded leaves (measurement location: 1/3, 1/2, and 2/3 of the distance from the leaves base) from the top of the plant were used for SPAD meter and Dualex measurements, and ten representative plants were randomly selected in each plot. All measurements were averaged to represent the leaf sensor data of each plot. The RS active sensor was held manually approximately 0.80 m above the canopy and at a constant speed in each plot (about 0.5 m s^−1^). The RS sensor measurement path was parallel to the plant row. Three rows of wheat were randomly selected to obtain the two default vegetation indices of NDRE ((NIR − RE)/(NIR + RE)) [30] and NDVI ((NIR − R)/(NIR + R)) [31], with average vegetation indices collected to represent the spectral data of each plot.

### 2.3. Plant Sampling and Measurements

The plots in the field experiments are often small (42, 30 and 63 m^2^ in Experiments 1, 2, and 3, respectively) and grown evenly; therefore, it is conventional to take representative samples and optical sensor measurements at different locations in each experimental plot [8,32]. Plant sampling was synchronously performed upon the completion of spectral measurements. Twenty wheat plants were randomly sampled and destructively separated into stems, leaves, and spikes. Each sub-sample was oven-dried at 105 °C for 30 min to stop all metabolic processes and samples were dried at 80 °C until reaching a constant weight. Samples were weighed and the leaf dry matter (LDM), stem dry matter (SDM), and spike dry matter (SpDM) were determined. The leaf N concentration (LNC), stem N concentration (SNC), and spike N concentration (SpNC) were measured using the standard Kjeldahl method [33].

LNA (kg ha^−1^) was used to measure N accumulation in the leaves (Equation (1)). PNA (kg ha^−1^) was calculated as the sum of leaf, stem, and spike N accumulation (Equation (2)). Plant N content (PNC (%); Equation (3)) was determined as the ratio of PNA (kg ha^−1^) and plant biomass (kg ha^−1^):

(1) LNA=LDM×LNC

(2) PNA=LDM×LNC+SDM×SNC+SpDM×SpNC

(3) $$\mathrm{PNC}=\frac{\mathrm{PNA}}{\mathrm{LDM}+\mathrm{SDM}+\mathrm{SpDM}}$$

The $N_{c}$ curve was employed as described by Jiang et al. [27]. The NNI could be calculated based on Equation (5):

(4) $$N_{c}=4.17\times W^{-0.39}$$

(5) $$\mathrm{NNI}=N_{a}/N_{c}$$

where W is the weight of the plant (Mg ha^−1^), Na is the actual plant N concentration, and Nc is the critical plant N concentration.

### 2.4. Data Analysis

Data obtained from experiments 1, 2, and 3 were used for the analysis of variance between the six sensor-based indices and N status parameters using SPSS 24 software. The exponential relationship between six sensor-based indices and LNC, LNA, PNC, PNA, and NNI were calibrated and validated with 10-fold cross-validation procedure based on the data from experiments 1–3. Model performance was evaluated using the coefficients of determination (R^2^), root mean square error (RMSE), and the relative error (RE (%)). The GraphPad Prism 8 software was used to plot the diagrams.

(6) $$\mathrm{RMSE}=\sqrt{\frac{1}{n}\times \sum_{i=1}^{n}{\left(P_{i}-O_{i}\right)}^{2}}$$

(7) $$\mathrm{RE}\left(\%\right)=100\times \sqrt{\frac{1}{n}\times \sum_{i=1}^{n}{\left(\frac{P_{i}-O_{i}}{O_{i}}\right)}^{2}}$$

where n is the number of samples, $O_{i}$ is the measured value, and $P_{i}$ is the predicted value.

N deficiency (NNI < 0.95), N optimal (0.95 ≤ NNI ≤ 1.05), and N excessive (NNI > 1.05) were used for diagnostic analysis [8,34]. The diagnostic category of predicted NNI using NBI and NDRE models was compared to those of observed NNI by areal agreement and the Kappa coefficient [35]. The areal agreement means the percentage of the two groups having same diagnostic category. The Kappa coefficient range of 0.21–0.40, 0.41–0.60, and 0.61–0.80 indicates fair, moderate, and substantial strength, respectively, of the diagnostic agreement [36]. N diagnostic maps were plotted using ArcGIS 10.3 software.

(8) $$\mathrm{Kappa}\;\mathrm{Coefficent}=\frac{\mathrm{Observed}\;\mathrm{Accuracy}-\mathrm{Chance}\;\mathrm{Agreement}}{1-\mathrm{Chance}\;\mathrm{Agreement}}$$

## 3. Results and Analysis

### 3.1. Variability of Nitrogen Status Indicators

Agronomic data from experiments 1, 2, and 3 were used for statistical analysis. The results showed that LNC, LNA, PNC, PNA, and NNI varied across growth stages, N levels, cultivars, and site-years (Table 3). LNA (coefficient of variation (CV) = 61.04%) was most variable across all growth stages, followed by PNA (CV = 50.16%), NNI (CV = 40.45%), and PNC (CV = 37.23%). LNC had a minimal CV of 26.44%. The analysis also indicated that the CV of the five N parameters were variable across growth stages. For example, the LNC was most variable at the jointing stage (CV = 30.10%), and had similar CV values (23.35–23.94%) to the other three growth stages. In contrast, the CV of PNC (27.59–34.36%), PNA (42.66–59.98%), and NNI (33.10–46.37%) gradually decreased as the growth stage progressed. The great variability of those N indicators will help to assess the ability of the optical sensors when monitoring and diagnosing wheat N status.

### 3.2. Dynamic Changes of Six Sensor-Based Indices under Different N Treatments

**Figure 3:**
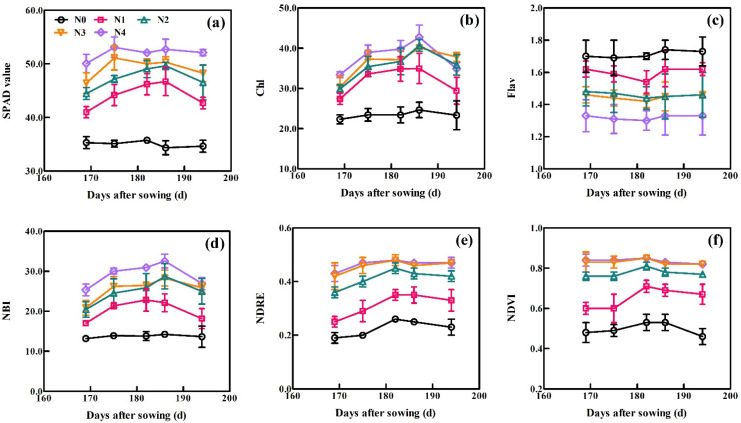
Dynamic variation in (**a**) SPAD, (**b**) chl, (**c**) Flav, (**d**) NBI, (**e**) NDRE, and (**f**) NDVI at the indicated days after sowing (DAS). Data were obtained from Experiment 1 using the XM30 cultivar. Vertical bars at each growth stage represent the standard error.

The dynamic changes in the six sensor-based indices with days after sowing (DAS) across all growth periods of XM30 in Experiment 1 are shown in Figure 3. All six sensor-based indices, excluding Flav, exhibited similar trends as the wheat growth progressed. These spectral indices treated with high N application rates generally exceeded these treated with low N. The SPAD initially increased and then gradually decreased under low N treatments (0, 90 and 180 kg N ha^−1^) as each growth stage progressed. Under conditions of high N application (270 and 360 kg N ha^−1^), the SPAD value rapidly increased and remained high before declining, indicating the SPAD values reached saturation. For Dualex, the values of Chl and NBI increased gradually, reached peak values at 186 DAS, and then decreased during plant aging. Trends for the Flav were the opposite of that for the Chl and NBI, where the Flav initially decreased and then increased after 186 DAS. For the RS sensor, the default vegetation indices of NDRE and NDVI increased slowly and then gradually declined. Curves at 270 and 360 kg N ha^−1^ applications were close to overlapping, indicating that the plant growth achieved a non-N limited status.

### 3.3. Relationship between the Six Sensors-Based Indices and Four N Indicators

Nitrogen indicators, such as LNC, LNA, PNC, and PNA, were collected in four spectral sensing stages: jointing, booting, flowering, and filling. Based on the data obtained from experiments 1–3, the quantitative exponential relationship between the six sensor-based indices and four N indicators were systematically analyzed, and the 10-fold cross-validation results were showed in Table 4. The results showed SPAD had R^2^ values of 0.25–0.60, 0.29–0.54, 0.28–0.57, and 0.23–0.54, RMSE values of 0.46–0.78, 17.69–29.85 kg ha^−1^, 0.24–0.55, and 41.29–52.96 kg ha^−1^, and RE values of 16.25–25.08%, 53.04–80.50%, 18.22–33.85%, and 56.56–82.88% for LNC, LNA, PNC, and PNA estimation, respectively, at single and all growth stages. The Chl index of Dualex had a similar performance to the SPAD value, with R^2^ values of 0.25–0.68, 0.27–0.52, 0.29–0.69, and 0.24–0.53, RMSE values of 0.44–0.79, 15.40–28.81 kg ha^−1^, 0.21–0.54, and 38.86–50.07 kg ha^−1^, and RE values of 17.20–22.44%, 54.64–81.25%, 17.93–32.28%, and 39.77–77.54% for LNC, LNA, PNC, and PNA estimation, respectively, at single and all growth stages. The NBI index performed relatively better than the other two Dualex-based indices (Chl and Flav) for estimating LNC (R^2^ = 0.36–0.79, RMSE = 0.39–0.67, RE = 13.43–20.02%), LNA (R^2^ = 0.49–0.70, RMSE = 14.35–23.50 kg ha^−1^, RE = 48.33–67.38%), PNC (R^2^ = 0.49–0.76, RMSE = 0.19–0.46, RE = 15.63–27.44%), and PNA (R^2^ = 0.53–0.72, RMSE = 34.63–39.32 kg ha^−1^, RE = 35.46–65.43%) at the single growth stage and across all growth stages. For the RS active canopy sensor, the default vegetation index of NDRE was more closely associated with LNC (R^2^ = 0.61–0.79, RMSE = 0.39–0.53, RE = 11.84–15.79%), LNA (R^2^ = 0.66–0.87, RMSE = 12.39–19.79 kg ha^−1^, RE = 24.72–42.12%), PNC (R^2^ = 0.51–0.74, RMSE = 0.18–0.45, RE = 14.81–27.34%), and PNA (R^2^ = 0.64–0.87, RMSE = 24.09–36.39 kg ha^−1^, RE = 20.56–36.21%) than NDVI at single growth stage and across all growth stages.

### 3.4. Relationship between the Optimal Index of Each Sensor and N Nutrition Index

**Figure 4:**
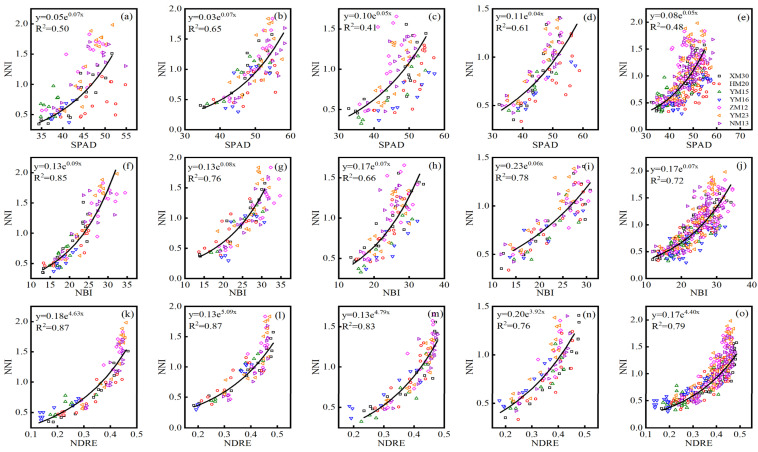
The exponential relationship between the SPAD and N nutrition index (NNI) across experiments 1–3 at (**a**) jointing, (**b**) booting, (**c**) flowering, (**d**) filling, and (**e**) all growth stages/the exponential relationship between the NBI and N nutrition index (NNI) across experiments 1–3 at (**f**) jointing, (**g**) booting, (**h**) flowering, (**i**) filling, and (**j**) all growth stages/the exponential relationship between the NDRE and N nutrition index (NNI) across experiments 1–3 at (**k**) jointing, (**l**) booting, (**m**) flowering, (**n**) filling, and (**o**) all growth stages. Black lines indicate regression lines.

The NBI index performed consistently well for the assessment of leaf (LNC and LNA) and plant (PNC and PNA) N status across three Dualex-based indices during all growth stages, and the default vegetation index NDRE of the RS sensor also displayed a consistently high correlation with LNC, LNA, PNC, and PNA. Hence, the optimal index (SPAD, NBI, and NDRE) of the SPAD meter, Dualex, and RS sensor were selected for establishing the relationship with NNI. Based on the data collected from experiments 1–3, the quantitative exponential relationship between the SPAD, NBI, NDRE, and NNI were systematically analyzed, and the 10-fold cross-validation results were showed in Table 5. The NDRE of the RS sensor performed best for monitoring NNI across different cultivars at jointing (R^2^ = 0.75–0.96, RMSE = 0.14–0.19, RE = 13.72–29.27%), booting (R^2^ = 0.73–0.97, RMSE = 0.09–0.24, RE = 10.04–24.18%), flowering (R^2^ = 0.76–0.86, RMSE = 0.12–0.20, RE = 12.19–24.69%), filling (R^2^ = 0.74–0.96, RMSE = 0.07–0.21, RE = 10.18–28.02%), and all (R^2^ = 0.67–0.87, RMSE = 0.12–0.26, RE = 18.53–24.39%) growth stages, followed by the Dualex-based index of NBI, with R^2^ of 0.53–0.88, 0.59–0.87, 0.33–0.87, 0.72–0.84, and 0.56–0.75, RMSE of 0.13–0.29, 0.11–0.33, 0.15–0.33, 0.13–0.19, and 0.16–0.29, RE of 19.33–36.40, 16.53–48.32, 16.74–52.83, 12.78–23.49, and 21.25–43.45 for NNI estimation across seven cultivars at jointing, booting, flowering, filling, and all growth stage, respectively. The SPAD had a slightly worse performance for estimating NNI at jointing (R^2^ = 0.29–0.81, RMSE = 0.16–0.48, RE = 19.65–77.65%), booting (R^2^ = 0.38–0.84, RMSE = 0.18–0.37, RE = 18.51–63.60%), flowering (R^2^ = 0.26–0.81, RMSE = 0.23–0.39, RE = 24.47–62.99%), filling (R^2^ = 0.48–0.87, RMSE = 0.13–0.28, RE = 15.45–45.56%), and all (R^2^ = 0.37–0.73, RMSE = 0.18–0.37, RE = 27.28–59.45%) growth stages. The seven cultivars performed differently at different growth stages, the XM30 had a consistent well validation results based on SPAD (R^2^ = 0.64–0.81, RMSE = 0.20–0.24, RE = 21.74–34.38%), NBI (R^2^ = 0.73–0.87, RMSE = 0.15–0.19, RE = 16.68–23.04%), and NDRE (R^2^ = 0.83–0.96, RMSE = 0.13–0.16, RE = 13.89–22.62%) at single and all growth stages. The YM15 performed poorly at jointing stage (R^2^ = 0.29–0.75, RMSE = 0.13–0.16, RE = 22.62–27.51%), while it achieved a relatively good performance at booting (R^2^ = 0.72–0.89, RMSE = 0.10–0.18, RE = 10.75–18.54%), flowering (R^2^ = 0.81–0.87, RMSE = 0.13–0.23, RE = 21.56–45.14%), and filling (R^2^ = 0.72–0.77, RMSE = 0.12–0.15, RE = 15.92–23.49%) stages among three optimal sensor indices. Therefore, the exponential relationship between the SPAD, NBI, NDRE, and NNI across seven cultivars using data from experiments 1–3 was constructed and shown in Figure 4, which had an R^2^ value of 0.41–0.65 for SPAD, 0.66–0.85 for NBI, and 0.76–0.87 for NDRE at single and all growth stages.

### 3.5. N Diagnosis of Winter Wheat Based on the SPAD, NBI, and NDRE at Different Growth Stages

To evaluate the diagnosis accuracy of the SPAD, NBI, and NDRE models, experimental plots in Experiment 1–3 were divided into three categories: N deficient (NNI < 0.95), N optimal (0.95 ≤ NNI ≤ 1.05), and N excessive (NNI > 1.05) based on the diagnosis threshold values. The results in Table 6 indicated that the diagnosis accuracy ranged from 0.54 to 0.71 for SPAD, 0.66 to 0.84 for NBI, and 0.72 to 0.86 for NDRE. The kappa coefficient ranged from 0.30 to 0.52 for SPAD, 0.42 to 0.72 for NBI, and 0.53 to 0.75 for NDRE across all growth stages. Based on the evaluation criteria, the NBI and NDRE models performed moderately well for the diagnosis of N status during each growth stage; the SPAD model performed moderately well at the jointing and booting stages, but had fair diagnosis agreement at the flowering and filling stages.

**Figure 5:**
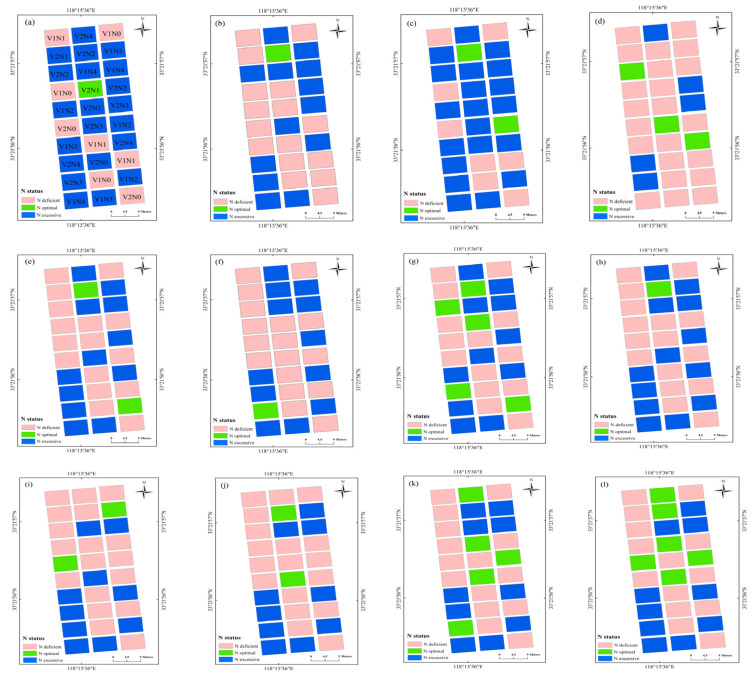
N diagnosis maps (experiment 1) based on the SPAD at the (**a**) jointing, (**b**) booting, (**c**) flowering, and (**d**) filling stage. N diagnosis maps based on the NBI at (**e**) jointing, (**f**) booting, (**g**) flowering, and (**h**) filling stage. N diagnosis maps based on the NDRE at (**i**) jointing, (**j**) booting, (**k**) flowering, and (**l**) filling stage. V1 and V2 in (**a**) represent XM30 and HM20 cultivars. N1, N2, N3, and N4 in (**a**) represent 0, 90, 180, 270, and 360 kg N ha^−1^ treatments, respectively, in Experiment 1.

The N diagnosis maps based on the SPAD, NBI, and NDRE models at each of the growth stages during 2016 are shown in Figure 5. The N diagnosis data of each field plot were variable across the growth stages, with a large variation in winter wheat N status observed at differing values of N application. The N diagnosis map based on NBI and NDRE had similar performance, which showed that experimental plots with 270 or 360 kg N ha^−1^ were well- or over-fertilized, which fall into the N optimal or N excessive categories, respectively, across different growth stages. In contrast, experimental plots with 0 or 90 kg N ha^−1^ showed deficient fertilization, falling into the N deficient category at each growth stage. The N diagnosis map based on SPAD showed a relatively worse diagnosis results, which classified several low N treatments (90 kg N ha^−1^) as N excessive category at the jointing and flowering stages, and classified several high N treatments (360 kg N ha^−1^) in the N-deficient category at the filling stage. Overall, the sensor values at each of the experimental plot showed varying crop growth and N status performance due to variable N rates.

## 4. Discussion

### 4.1. Wheat N Status Assessments Based on the Leaf and Canopy Sensors

The SPAD meter consists of a single leaf spectrometer and uses a red spectrum at 650 nm to estimate the chlorophyll concentration in the leaves, which significantly correlates with crop N status [13]. In Figure 3a, the SPAD value increased as wheat growth progressed, then remained high under high N levels (270 and 360 kg N ha^−1^), which may have a saturated or near-saturated status in conditions of high N supply. Yue et al. [14] indicated that the response of the SPAD readings to wheat PNC showed a saturated phenomenon with an N supply that gradually increased to excessive amounts, i.e., to 420 kg N ha^−1^, consistent with our study. The Dualex uses two near-infrared (710 and 850 nm) bands to estimate the chlorophyll content. Chlorophyll fluorescence (375 nm) of the sensor can be used to monitor leaf flavonoid content [37]. Zhang et al. [17]. showed that the Dualex (R^2^ = 0.87) outperformed the SPAD meter for estimations of rice chlorophyll (R^2^ = 0.77), and could mitigate the influence of saturating conditions under high N concentrations. Figure 3c showed wheat crops under N deficiency accumulate higher levels of Flav, the response of which contrasts Chl (Figure 3b). Gabriel et al. [22]. indicated that complementary polyphenol information (as Flav) can improve maize N deficiency predictions. The NBI index performed relatively better than the other two Dualex-based indices (Chl and Flav) for LNC (Table 4: R^2^ = 0.36–0.79, RMSE = 0.39–0.67, RE = 13.43–20.02%), LNA (Table 4: R^2^ = 0.49–0.70, RMSE = 14.35–23.50 kg ha^−1^, RE = 48.33–67.38%), PNC (Table 4: R^2^ = 0.49–0.76, RMSE = 0.19–0.46, RE = 15.63–27.44%), and PNA (Table 4: R^2^ = 0.53–0.72, RMSE = 34.63–39.32 kg ha^−1^, RE = 35.46–65.43%) estimation across single and all growth stages. The NBI can partially alleviate the influence of gradients along the plant leaf, and can accentuate the difference amongst the levels of plant N deficit due to the inverse dependence between Chl and Flav on the plant N nutritional status [38]. Previous studies have also shown that the NBI performed well for the assessment of N status and growth in muskmelon, consistent with this study [39].

The measured area of the SPAD meter was 6 mm^2^ and the sampling area of each plot was 540 mm^2^ based on our measurement method (90 individual measurements in each experiment plot). For Dualex, the sampling area was 1800 mm^2^ (the measured area was 20 mm^2^). The smaller sampling area for the SPAD meter was susceptible to factors such as blade veins and internal variation of the field. Accordingly, the SPAD values were less accurate for the estimation of crop N nutrition. The field of view of the RS sensor is 45° by 10° and the scan width of sensor perpendicular to the flight direction is 0.66 m (measurement height of 0.80 m). As a result, the scan area was a minimum of 5.94 square meters for each plot (e.g., Experiment 3). The larger sampling area in each plot was more representative of crop growth status. The NDRE derived from the RS sensor was closely associated with LNC (Table 4: R^2^ = 0.61–0.79, RMSE = 0.39–0.53, RE = 11.84–15.79%), LNA (Table 4: R^2^ = 0.66–0.87, RMSE = 12.39–19.79 kg ha^−1^, RE = 24.72–42.12%), PNC (Table 4: R^2^ = 0.51–0.74, RMSE = 0.18–0.45, RE = 14.81–27.34%), and PNA (Table 4: R^2^ = 0.64–0.87, RMSE = 24.09–36.39 kg ha^−1^, RE = 20.56–36.21%) at single growth stage and across all growth stages. Other studies indicated that the active canopy sensor (r = 0.73–0.86) displayed a higher accuracy in predicting the grapevine biomass and yield compared to the chlorophyll meter (r = 0.62–0.76) [40], which is consistent with this study.

### 4.2. Wheat N Nutrition Diagnosis Based on the Optimal Indices (SPAD, NBI, and NDRE) of Three Sensors

The estimation and diagnosis of N nutrition is a key consideration in precision wheat management [41]. NNI acts as an optimal N diagnostic indicator, with the development of remote sensing technologies making real-time estimations of NNI possible. In this study, the NNI diagnosis models based on the optimal indices (SPAD: R^2^ = 0.41–0.65; NBI: R^2^ = 0.66–0.85; NDRE: R^2^ = 0.76–0.87) of three sensors were conducted during each growth stage, permitting the N diagnosis at all stages of wheat growth development. During the construction of the model, the R^2^ of the relationship between NNI and NDRE at the flowering (Figure 4: R^2^ = 0.83) and filling (Figure 4: R^2^ = 0.76) stages were slightly lower than during the jointing (Figure 4: R^2^ = 0.87) and booting (Figure 4: R^2^ = 0.87) stages, which may be due to the emergence of a spike that influenced the estimated performance of the NDRE models at the late growth period. Similar results have also been shown in studies of rice and wheat [28,42]. The 10-fold cross-validation results of the relationship between three optimal indices and NNI across seven wheat cultivars showed that the Dualex-based NBI performed better than SPAD, and NDRE of RS canopy sensor performed better than two leaf-sensor indices at single and all growth stages. The seven wheat cultivars performed differently at different stage, which may be due to the difference of variety characteristics and climatic condition among different years [43,44]. The XM30 had a consistent good performance at each growth stage, the seedling of XM30 half creep flat on the ground with dark green and board leaves, which had a strong tilling ability and relative loose plant type. Winter wheat N status can be diagnosed based on NNI predictions using established diagnostic models and NNI threshold values. The NBI model (Table 6) had an areal agreement of 0.66 to 0.84 and a kappa coefficient of 0.42 to 0.72 across different growth stages. Similar studies have shown that the NBI measured using a Multiplex 3 sensor performs well for the diagnosis of rice N status during stem elongation (areal agreement = 75%, kappa coefficient = 0.595) and heading (areal agreement = 80%, kappa coefficient = 0.590) stages [45]. The NDRE model (Table 6: areal agreement = 0.72–0.86, kappa coefficient = 0.53–0.75) based on the RS sensor performed to a relatively higher level than the SPAD and NBI model, which also indicated that the larger sampling area by the canopy optical sensor was considerably more representative for wheat growth status [39]. Other studies have shown that the NDRE model based on the RS sensor can accurately measure rice N status at the panicle initiation and jointing stage (areal agreement = 0.55, kappa coefficient = 0.30) and heading growth stage (areal agreement = 0.76, kappa coefficient = 0.54) [46].

The N diagnosis plot maps based on the spectral indices directly reflected the N status of each plot (Figure 5), which may assist farmer for precise crop N management. As an example, the N diagnosis map and NNI diagnosis model could be coupled with the fertilization topdressing model to consider both temporal and spatial variability of crop growth and N nutrition [47,48], after which the N management decisions could be optimized to improve N use efficiency and increase economic benefits [49]. The spectral sensing method could non-destructively quantify and visualize the real time crop growth status compared to traditional leaf color-based diagnosis and chemical diagnosis, such as Kjeldahl digestion, which required laborious and time-consuming preparation [37]. The handheld spectral sensing used in this study was relatively limited when used for data collection in a large area compared to drone- and tractor-based sensing; the tractor-based Yara N-sensor was successfully used to estimate maize aboveground N uptake (R^2^ = 0.57–0.84) and dry matter yields (R^2^ = 0.67–0.91) at different growth stages [50]. Argento et al. [51] used the spectral index of NDRE derived from a UAV-mounted multispectral camera to guide N fertilizer application for winter wheat in a 2-hectare area, and to evaluate the sensitivity of NDRE with wheat dry matter (R^2^ = 0.72), NNI (R^2^ = 0.75) across different growth stages. In addition, the combination of ground and areal remote sensing data may be a promising method in crop growth monitoring, Zheng et al. [52] demonstrated the integration (R^2^ = 0.72–0.75) of ground-based narrow band vegetation indices with UAV-based textural information exhibited a significant improvement for rice PNC estimation compared to individual UAV data (R^2^ = 0.59–0.70). Due to the time and labor required for synchronous sensor data and agronomic indicators, this study analyzed growth stages that were initiated at the jointing stage. Monitoring frequency prior to the jointing stage can be increased in future research to improve these diagnosis models. In addition, similar studies should be performed for different wheat cultivars in other eco-sites, further enhancing the practicability of the optical sensors during field production.

## 5. Conclusions

Our results demonstrate that three portable optical sensors (SPAD meter, Dualex, and RS sensor) could be used to estimate and diagnose the N status of wheat. The Dualex-based NBI had a relatively well performance among four leaf-sensor indices, while NDRE of RS sensor performed best for LNC, LNA, PNC, and PNA estimation across different growth stages due to larger sampling area of canopy sensor. The areal agreement of the NNI diagnosis models ranged from 0.54 to 0.71 for SPAD, 0.66 to 0.84 for NBI, and 0.72 to 0.86 for NDRE, and kappa coefficient ranged from 0.30 to 0.52 for SPAD, 0.42 to 0.72 for NBI, and 0.53 to 0.75 for NDRE across all growth stages. We conclude that the use of sensor-based diagnostic models is appropriate for the rapid and non-destructive diagnosis of N nutrition of winter wheat.

## Figures and Tables

**Table 1:** Basic information about the three field experiments.

Experiment No. Year	Location	Plot Size (m^2^)	Cultivar	N Rate (kg ha^−1^)	Sampling Stage (Date)
1 2015–2016	Sihong (33.37° N,118.26° E)	42 (6 m × 7 m)	Xumai30 (XM30) Huaimai20 (HM20)	0 90 180 270 360	Jointing (5 April) Booting (15 April) Heading (22 April) Flowering (26 April) Filling (4 May)
2 2016–2017	Rugao (32.27° N, 120.75° E)	30 (5 m × 6 m)	Yangmai15 (YM15) Yangmai16 (YM16)	0 150 300	Jointing (27 March) Booting (11 April) Flowering (22 April) Filling (7 May)
3 2017–2018	Xinghua (33.08° N, 119.98° E)	63 (7 m × 9 m)	Zhenmai12 (ZM12) Yangmai23 (YM23) Ningmai13 (NM13)	0 90 180 270 360	Jointing (9 April) Booting (15 April) Flowering (24 April) Filling (9 May)

**Table 2:** Characteristics of the three optical sensors.

Sensor Information	Chlorophyll Meter	Fluorescence Sensor	Reflectance Sensor
Sensor name	SPAD-502	Dualex 4 Scientific+	RapidScan CS-45
Manufacturer	Minolta Camera Co. (Osaka, Japan)	Force-A (Orsay, France)	Holland Scientific (Lincoln, NE, USA)
Measurement scale	Leaf	Leaf	Canopy
Field of view	-	-	10°–45°
Working height	-	-	0.3–3.0 m
Measurement area	6 mm^2^	20 mm^2^	Dependent on measurement height
Measuring Principle	Transmittance	Fluorescence	Reflectance
Spectral band	Red (650 nm) and near infrared (940 nm)	UV (375 nm), red (655 nm), red-edge (710 nm), and near infrared (850 nm)	Red (670 nm), red-edge (730 nm), and near infrared (780 nm)
Output parameter	SPAD value	Chl, Flav, NBI	Reflectance (670, 730, 780 nm); NDRE, NDVI
Abbreviation	SPAD meter	Dualex	RS sensor

**Table 3:** Descriptive statistics of leaf N concentration (LNC), leaf N accumulation (LNA), plant N concentration (PNC), plant N accumulation (PNA), and N nutrition index (NNI) across all growth stages.

Parameter	Growth Stage	N	Min.	Max.	SD ^a^	CV ^b^ (%)
LNC (%)	Jointing	93	1.78	5.22	1.03	30.10
	Booting	93	2.14	5.39	0.84	23.35
	Flowering	93	2.01	5.32	0.86	23.90
	Filling	93	1.59	4.31	0.72	23.94
	All	372	1.59	5.39	0.90	26.44
LNA (kg ha^−1^)	Jointing	93	8.64	158.33	40.01	64.23
	Booting	93	11.04	156.86	36.02	57.39
	Flowering	93	11.55	123.44	26.75	50.93
	Filling	93	5.51	90.58	22.23	55.39
	All	372	5.51	144.86	33.22	61.04
PNC (%)	Jointing	93	1.06	3.50	0.71	34.36
	Booting	93	0.85	3.17	0.62	32.23
	Flowering	93	0.71	2.61	0.50	31.34
	Filling	93	0.68	2.04	0.36	27.59
	All	372	0.68	3.50	0.64	37.23
PNA (kg ha^−1^)	Jointing	93	15.39	257.46	61.75	59.98
	Booting	93	21.27	274.88	58.92	51.29
	Flowering	93	28.63	276.51	57.71	46.14
	Filling	93	33.48	268.35	57.07	42.66
	All	372	15.39	276.51	59.77	50.16
NNI	Jointing	93	0.34	1.92	0.45	46.37
	Booting	93	0.30	1.84	0.40	40.89
	Flowering	93	0.33	1.65	0.34	37.20
	Filling	93	0.34	1.41	0.28	33.10
	All	372	0.30	1.92	0.38	40.45

^a^ SD indicates the standard deviation of the mean. ^b^ CV indicates the coefficient of variation (%).

**Table 4:** Ten-fold cross-validation results for the pair-wise exponential relationship between the six sensor-based indices and leaf N concentration (LNC), leaf N accumulation (LNA), plant N concentration (PNC), and plant N accumulation (PNA) at different growth stages across experiments 1–3.

Parameter	Sensor	Index	Jointing Stage			Booting Stage			Flowering Stage			Filling Stage			All Stage		
			R^2^	RMSE	RE (%)	R^2^	RMSE	RE (%)	R^2^	RMSE	RE (%)	R^2^	RMSE	RE (%)	R^2^	RMSE	RE (%)
LNC (%)	SPAD	SPAD	0.44	0.77	25.08	0.59	0.53	16.25	0.25	0.78	21.68	0.60	0.46	16.28	0.39	0.70	21.21
	Dualex	NBI	0.79	0.47	13.43	0.66	0.50	14.48	0.36	0.67	20.02	0.71	0.39	14.19	0.61	0.56	16.74
		Chl	0.68	0.58	18.66	0.57	0.55	16.27	0.25	0.79	22.44	0.65	0.44	17.20	0.42	0.68	20.00
		Flav	0.66	0.60	19.21	0.43	0.63	19.22	0.27	0.74	21.34	0.50	0.50	19.24	0.51	0.62	20.70
	RS	NDRE	0.79	0.47	15.02	0.75	0.42	11.84	0.61	0.53	15.79	0.70	0.39	13.80	0.70	0.49	15.03
		NDVI	0.72	0.55	18.61	0.56	0.55	17.05	0.56	0.57	17.86	0.62	0.44	19.45	0.64	0.54	19.03
LNA (kg ha^−1^)	SPAD	SPAD	0.36	29.85	80.50	0.54	24.86	67.24	0.29	24.32	62.51	0.46	17.69	53.04	0.29	29.11	78.15
	Dualex	NBI	0.70	23.50	67.38	0.63	22.31	52.52	0.49	18.47	55.47	0.58	14.35	48.33	0.53	23.46	59.49
		Chl	0.51	28.81	81.25	0.50	26.12	69.43	0.27	24.73	69.82	0.52	15.40	54.64	0.31	28.51	78.51
		Flav	0.50	30.23	83.25	0.49	28.32	70.21	0.49	18.95	58.39	0.50	15.55	60.67	0.53	23.43	68.19
	RS	NDRE	0.87	15.04	24.72	0.77	17.71	24.75	0.78	12.39	24.81	0.66	13.62	42.12	0.67	19.79	40.55
		NDVI	0.86	16.27	37.82	0.68	20.55	37.09	0.74	13.65	33.63	0.66	15.49	44.25	0.63	21.43	42.00
PNC (%)	SPAD	SPAD	0.36	0.53	31.75	0.50	0.44	26.44	0.30	0.42	29.56	0.57	0.24	18.22	0.28	0.55	33.85
	Dualex	NBI	0.76	0.36	17.22	0.65	0.36	21.85	0.50	0.35	24.04	0.70	0.19	15.63	0.49	0.46	27.44
		Chl	0.64	0.44	21.24	0.51	0.43	26.02	0.29	0.42	29.24	0.69	0.21	17.93	0.29	0.54	32.28
		Flav	0.61	0.45	24.84	0.47	0.45	29.61	0.32	0.41	32.47	0.44	0.27	22.63	0.45	0.47	31.85
	RS	NDRE	0.72	0.39	21.73	0.74	0.32	18.41	0.73	0.26	18.35	0.74	0.18	14.81	0.51	0.45	27.34
		NDVI	0.57	0.48	28.37	0.55	0.41	27.36	0.50	0.35	27.65	0.50	0.25	22.97	0.47	0.47	32.41
PNA (kg ha^−1^)	SPAD	SPAD	0.36	52.96	82.88	0.54	41.29	58.97	0.23	50.33	65.98	0.34	46.28	56.56	0.30	49.14	80.28
	Dualex	NBI	0.72	35.06	65.43	0.67	34.63	44.69	0.53	39.32	38.80	0.57	37.34	35.46	0.59	39.32	57.96
		Chl	0.50	46.95	77.54	0.52	41.78	59.44	0.24	50.07	56.16	0.53	38.86	39.77	0.38	47.87	74.69
		Flav	0.50	46.22	78.85	0.52	41.89	63.11	0.44	43.10	51.64	0.48	41.07	39.66	0.48	44.09	72.33
	RS	NDRE	0.87	24.09	20.56	0.75	30.26	24.00	0.76	27.96	21.18	0.64	36.39	36.21	0.68	34.72	32.53
		NDVI	0.82	28.22	33.07	0.65	35.41	34.41	0.57	37.59	33.69	0.62	37.24	37.99	0.62	37.50	36.91

**Table 5:** Ten-fold cross-validation results for the pair-wise exponential relationship between the SPAD, NBI, NDRE, and NNI across seven cultivars at different growth stages in experiment 1–3.

Index	Cultivar	Jointing Stage			Booting Stage			Flowering Stage			Filling Stage			All Stage		
		R^2^	RMSE	RE (%)	R^2^	RMSE	RE (%)	R^2^	RMSE	RE (%)	R^2^	RMSE	RE (%)	R^2^	RMSE	RE (%)
SPAD	XM30	0.81	0.22	34.38	0.70	0.22	21.74	0.64	0.24	24.47	0.76	0.20	26.01	0.73	0.21	31.36
	HM20	0.31	0.48	77.65	0.41	0.32	52.62	0.58	0.28	48.29	0.48	0.28	45.56	0.48	0.33	55.81
	YM15	0.29	0.16	27.51	0.72	0.18	18.54	0.81	0.23	45.14	0.77	0.13	20.78	0.63	0.18	32.28
	YM16	0.32	0.22	52.65	0.45	0.27	63.60	0.78	0.36	62.99	0.78	0.20	29.82	0.56	0.29	59.45
	ZM12	0.67	0.42	27.09	0.84	0.25	18.51	0.36	0.39	29.42	0.78	0.17	15.45	0.44	0.37	27.28
	YM23	0.79	0.42	29.93	0.38	0.37	29.04	0.26	0.27	26.18	0.87	0.20	17.45	0.42	0.37	27.91
	NM13	0.50	0.28	19.65	0.68	0.24	29.45	0.56	0.28	29.08	0.78	0.14	15.67	0.41	0.28	27.95
	All varieties	0.40	0.36	48.06	0.53	0.27	35.75	0.35	0.30	38.68	0.50	0.20	26.44	0.37	0.30	38.41
NBI	XM30	0.83	0.18	23.04	0.87	0.15	16.68	0.75	0.18	16.74	0.77	0.15	18.94	0.73	0.19	21.56
	HM20	0.85	0.13	19.35	0.64	0.18	23.03	0.78	0.15	22.58	0.78	0.13	20.76	0.74	0.15	21.90
	YM15	0.55	0.13	24.46	0.87	0.11	16.53	0.87	0.20	40.36	0.72	0.15	23.49	0.75	0.16	28.27
	YM16	0.79	0.17	36.40	0.54	0.24	48.32	0.75	0.33	52.83	0.77	0.13	18.37	0.58	0.25	43.45
	ZM12	0.66	0.29	21.47	0.76	0.23	22.69	0.49	0.28	21.96	0.78	0.13	12.78	0.64	0.24	21.25
	YM23	0.88	0.24	20.47	0.59	0.33	25.54	0.33	0.27	24.07	0.84	0.19	16.18	0.65	0.29	23.27
	NM13	0.53	0.28	19.33	0.76	0.19	20.40	0.58	0.26	24.52	0.78	0.13	14.19	0.56	0.24	22.46
	All varieties	0.78	0.22	23.12	0.71	0.22	27.03	0.54	0.23	28.44	0.72	0.15	17.73	0.65	0.23	26.43
NDRE	XM30	0.96	0.14	13.94	0.95	0.13	13.89	0.83	0.15	17.53	0.85	0.16	22.62	0.87	0.16	18.53
	HM20	0.87	0.18	19.91	0.73	0.17	19.43	0.81	0.13	15.62	0.74	0.17	28.02	0.75	0.16	21.44
	YM15	0.75	0.15	22.62	0.89	0.10	10.75	0.86	0.13	21.56	0.76	0.12	15.92	0.72	0.16	24.39
	YM16	0.83	0.15	29.27	0.97	0.09	10.04	0.83	0.14	24.69	0.96	0.07	10.18	0.85	0.12	20.12
	ZM12	0.91	0.18	15.28	0.77	0.22	20.23	0.76	0.20	16.75	0.75	0.17	15.22	0.71	0.24	20.68
	YM23	0.93	0.19	13.72	0.74	0.24	18.44	0.84	0.12	12.19	0.83	0.21	18.15	0.67	0.26	19.93
	NM13	0.81	0.17	16.47	0.79	0.21	24.18	0.79	0.15	15.26	0.86	0.10	11.66	0.70	0.20	20.47
	All varieties	0.87	0.17	18.48	0.79	0.18	18.11	0.81	0.15	17.31	0.79	0.14	16.82	0.73	0.20	20.66

**Table 6:** Areal agreement and kappa coefficient for SPAD, NBI, and NDRE at different growth stages.

Index	Areal Agreement				Kappa Coefficient			
	Jointing	Booting	Flowering	Filling	Jointing	Booting	Flowering	Filling
SPAD	0.71	0.70	0.54	0.61	0.52	0.50	0.30	0.34
NBI	0.84	0.77	0.66	0.71	0.72	0.61	0.42	0.49
NDRE	0.86	0.84	0.80	0.72	0.75	0.69	0.65	0.53
